# Acupressure the PC6 point for alleviating postoperative nausea and vomiting: A systematic review protocol

**DOI:** 10.1097/MD.0000000000016857

**Published:** 2019-08-16

**Authors:** Jiao Yang, Yilu Jiang, Ying Chen, Mingsheng Sun, Jiao Chen, QianHua Zheng, Fan-rong Liang

**Affiliations:** Chengdu University of Traditional Chinese Medicine, Chengdu, China.

**Keywords:** acupressure, PC6 point, PONV, protocol, systematic review

## Abstract

**Background:**

Postoperative nausea and vomiting (PONV) are common complications following surgery and anesthesia, conventional drugs can carry some side effect in treating PONV. Acupressure PC6 point has been widely used in clinical, but there still exist controversy towards its effectiveness and safety. We, therefore, design this study to systematically assess the effectiveness and safety of acupressure PC6 point for treating PONV.

**Methods and analysis:**

Nine online databases will be searched from their inception to May 2019. We will include randomized controlled trials (RCTs) involving patients with PONV and receiving acupressure PC6 point treatment. Two independent reviewers will be responsible for the selection of studies, data extraction and risk of bias assessment. RevMan V.5.3 software will be used for data synthesis with either a fixed effects model or random effects model depending on the heterogeneity test. Evidence quality will be evaluated using the Grading of Recommendations Assessment, Development and Evaluation system (GRADE). The primary outcome is incidence of postoperative nausea (PON), postoperative vomiting (POV) and PONV events during 0 to 6 hours and after 6 hours of the treatment. The secondary outcome is the number of people who use emergency drugs and the number of people with adverse reactions. A meta-analysis will be conducted if no considerable heterogeneity is detected. The results will be presented as risk ratios with 95% confidence interval (CIs) for dichotomous data and weighted mean differences or standardized mean differences with 95% CIs for continuous data.

**Results:**

This study will provide a high-quality evidence to assess the effectiveness and safety of acupressure PC6 point for patient with PONV.

**Conclusion:**

This review will provide up-date evidence of whether acupressure of PC6 point is an effective and safe intervention for PONV. PROSPERO registration number: CRD42019135598

## 1 Introduction

Postoperative nausea and vomit (PONV) is one of the most common complications after surgery and anesthesia. Although it is not a life threatening complication, it can cause dehydration, electrolyte imbalance, wound dehiscence, pulmonary aspiration, and delayed hospital discharge.^[1,2,3]^ The general incidence of vomiting and nausea after surgery and anesthesia is about 30% and 50%, respectively.^[4]^ And the PONV rate can be as high as 80% in a subset of high-risk patients. It is reported that PONV can also result in a significant increase in overall health care costs.^[4]^ Despite the widespread use of antiemetic drugs, the management of PONV is unsatisfied, and PONV still affects about 20% to 40% of surgical patients after the use of drugs. Meanwhile, antiemetic drugs can also cause some side effects like sedation, headache, constipation, and fatigue.^[5]^ Therefore, anesthetists try to find some inexpensive and non-invasive methods to treat PONV.

Acupressure is a non-invasive therapeutic method applying physical pressure to certain acupuncture points by finger, elbow, hand or with various devices.^[6,7,8,9,10]^ The popularity of acupressure has increased over recent years and has been the fourth preferred complementary and alternative therapy in hospitalized patients in Australia,^[11]^ when most acupressure studies have focused on stimulating the ‘Pericardium (PC6) acupoint’ to reduce nausea and vomiting. Previous randomized controlled trials (RCTs) involving acupressure of PC6 point for treating PONV describe diverse clinical outcomes. Moreover, the systematic reviews^[12,13]^ were published before Jun 2013 reporting controversial results. It has been noticed that there were at least 7 RCTs has been published after 2013.^[7,14,15,16,17,18,19]^ These recent publications will be potential contributors to change the existing evidence. Therefore, we have an opportunity to re-evaluate the effectiveness and safety of acupressure PC6 point for patients with PONV.

Hence, we will perform a systematic review to evaluate the effectiveness and safety of acupressure PC6 point for patients with PONV. In addition, we will compare the effectiveness and safety of acupressure PC6 point with drugs, placebo, and other treatments. Moreover, we will assess when acupressure should be initiated and the duration of each session to achieve maximum antiemetic effect, as well as to draw scientific conclusions and further improve the application of acupressure PC6 point in PONV.

## 2 Methods

### 2.1 Inclusion criteria for study selection

#### 2.1.1 Types of studies

We will include RCT that were reported in English or Chinese. There is no limitation to the type of surgery and anesthesia. Non-RCTs reviews, quasi-RCTs, crossover trials, case report, animal experimental studies, expert experience, conference article and duplicated publications will be excluded. Any study with a sample size of less than 10 subjects will also be excluded.

#### 2.1.2 Types of patients

All eligible study participants will be included in this review regardless of the type of anesthesia or surgery, risk score for PONV, duration of anesthesia, nationality, gender, race, occupation, or education. Trials will include participants with American Society of Anesthesiologists (ASA) physical status classified as I-III. Trials including study participants who are not appropriate to receive acupressure therapy, such as pregnant or lactating women and those had any disease of the hand or wrist (such as a rash, local infection, burns, thromboendarteritis, tumor, or neuropathy) will be excluded.

#### 2.1.3 Types of interventions

##### 2.1.3.1 Experimental interventions

The interventions considered in the studies can be finger acupressure or wristband acupressure on PC6 acupoint. There was no restriction on the duration and frequency of PC6 acupoint acupressure or when it was applied.

##### 2.1.3.2 Control interventions

The patients who accept conventional drugs or sham/placebo acupressure would be included by control group

The following treatment comparisons will be investigated:

(a) Acupressure vs conventional drugs
(b) Acupressure vs placebo/sham acupuncture
(c) Acupressure combine with other therapy vs the same other therapy alone.

Studies that compare different forms of acupressure or compare acupressure with other complementary and alternative therapeutic interventions shall be excluded.

#### 2.1.4 Types of outcome measures

##### 2.1.4.1 Primary endpoints

The primary endpoints will be the incidences of PON, POV, and PONV during 0 to 6 hours and after 6 hours of the treatment.

##### 2.1.4.2 Secondary endpoints

The secondary outcomes is the number of people who use emergency drugs (such as Ondansetron and Metoclopramide) and the number of people who were reported with side effect caused by antiemetic drugs or PC6 acupoint acupressure, or both.

### 2.2 Search methods

The following databases will be searched from their inception to May 2019: MEDLINE, EMBASE, Cochrane Library, Chinese National Knowledge Infrastructure (CNKI), Chinese Biomedical Literature Database (CBM), Wanfang Database, the Chongqing VIP Chinese Science, and Technology Periodical Database (VIP). World Health Organization Clinical Trials Registry, ClinicalTrials.gov. Reference lists of relevant articles, reviews, and trials.

The following medical search headings (MeSH) will be used: postoperative complications, nausea and vomiting, acupressure, manipulation, complementary therapies, shiatsu, wristband, finger pressure, randomized controlled trial, randomized controlled, randomized, controlled, and clinical trial. Corresponding Chinese words of the aforementioned search terms will be used for the Chinese databases. The searching strategy for MEDLINE is listed in Table 1.

**Table 1:**
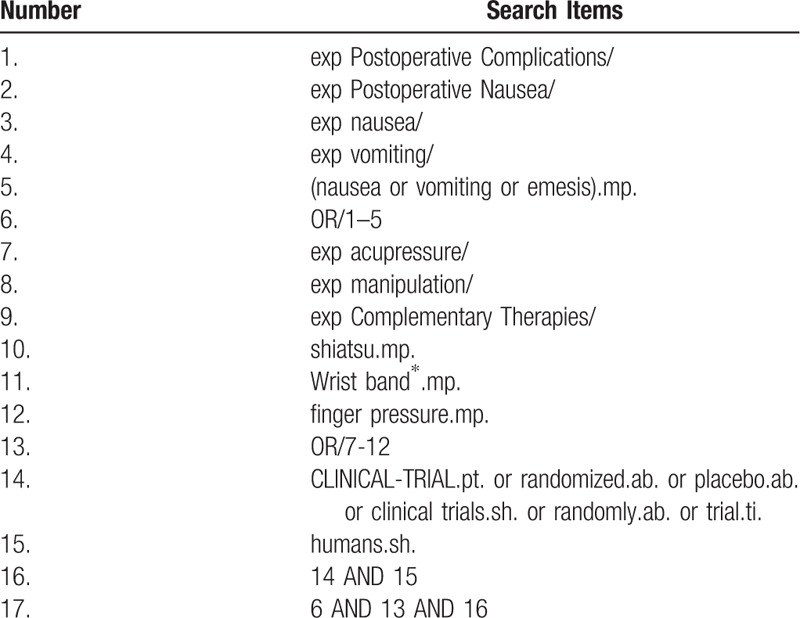
Search strategy in MEDLINE (Ovid SP).

### 2.3 Data collection and analysis

#### 2.3.1 Selection of studies

**Figure 1:**
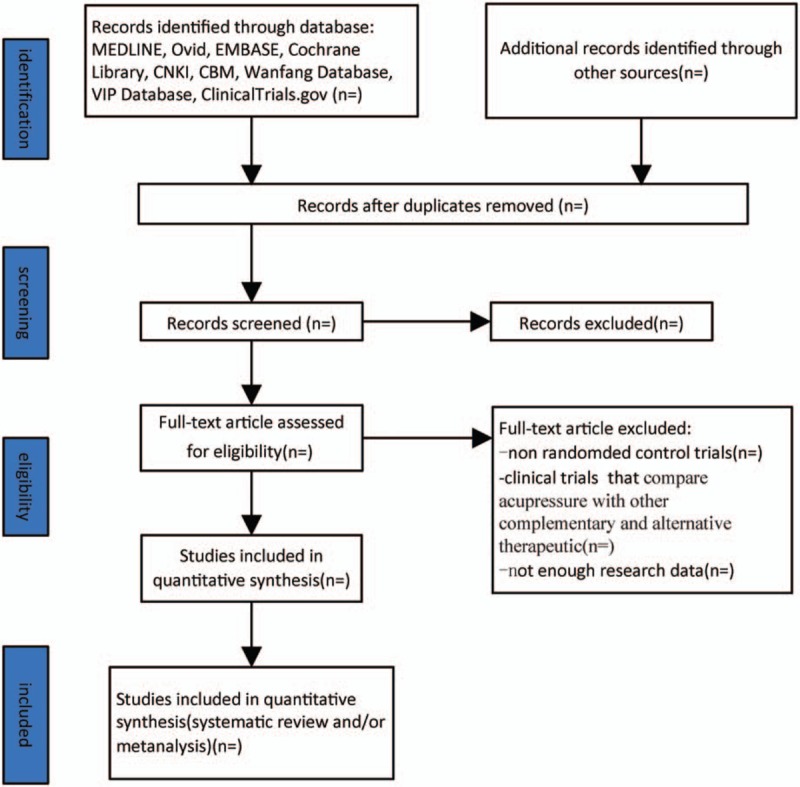
Illustrates the flow diagram of studies identified.

All reviewers are trained to ensure a good understanding of the purpose and process of the review. The search results will be imported from the original databases into Endnote X9. Duplicates studies will be removed, 2 reviewers (JY and YLJ) will preliminary filter the article by screening the titles, abstracts, and keywords. Duplicated and ineligible mismatched research will be removed. The cause of the exclusion will be recorded as an Excel data set. The next step will be to further evaluate the studies that meet the inclusion criteria by reading the full text and fabrication extract form for study details. The list of studies will be checked by the 2 reviewers (YJ and YLJ) to identify trials that may be missed. And the selection results will be cross-checked by the 2 reviewers. Any disagreement will be resolved by consensus. Further debate will be arbitrated by the third reviewer (QHZ). Each eligible trial will be assigned a study ID format as follows: surname of the first author + space + year of publication (e.g., Zheng, 2018). Study selection is summarized in a PRISMA flow diagram (Fig. 1).

#### 2.3.2 Data collection and management

Two reviewers (MSS and YC) will independently extract data from the selected studies, and any disagreement will be resolved through discussions or negotiation with a senior reviewer (QHZ). We will extract the following information: the title of journals, year of publication, author list, type of randomization, type and duration of anesthesia and surgery, type of acupressure, type of control, sample size, details of participants, timing and technique of intervention, frequency and duration of intervention, results, conclusion, side effects, and the use of rescue therapy.

#### 2.3.3 Assessment of risk of bias in included studies

The risk of bias will be evaluated using the Cochrane Collaboration's tool for assessing risk of bias in randomized trials.^[20]^ Two reviewers (YJ and YLJ) will input the relevant information of each trial into the RevMan software (V5.3) and evaluate the trial for at least 6 domains (random sequence generation, assignment concealment, blinding of participants and personnel, blinding of outcome assessment, incomplete outcome data, selective reporting and other biases if necessary). For each domain, the trial will be rated as high, unclear or low risk bias. Trials that are rated as high risk in one or more domains will be rated as ‘high risk’, and trials that are rated low risk in all domains will be rated as ‘low risk’. If there is a low or unclear risk of bias for all key domains, the trial will be rated as ‘unclear risk’. If basic information is missing, there is a risk of bias assessment. We will contact the person or correspondent. The rating results will be cross-checked and the differences will be resolved through discussion and arbitration by the third reviewer (QHZ).

#### 2.3.4 Measures of treatment effect

Data will be synthesized and statistically analyzed in RevMan V.5.3. For dichotomous data, we will use a risk ratio with 95% confidence intervals (CIs) for analysis. For continuous data, we will use a weighted mean difference (WMD) or a standard mean difference (SMD) with 95% CIs for analysis. The WMD will be used for the same scale or same assessment instrument; SMD will be used for different assessment tools.

#### 2.3.5 Unit of analysis issues

Convert the units of each result from different trials to international units before performing statistical analysis.

#### 2.3.6 Dealing with missing data

In the case of missing data, we will consider why data is lost (randomly or not). Whenever possible, we will try to contact the original investigator and ask them to provide any insufficient and missing data for inclusion in the study. If the missing data is not available, an available case study will be performed (only data with known results). And we will discuss the potential impact of missing data on the review results in the discussion section.

#### 2.3.7 Assessment of heterogeneity

Χ^2^ test will be performed to investigate the statistical heterogeneity before statistical analysis. If the resulting *P* value < .10, it indicates significant heterogeneity of the test. Moreover, the I^2^ value will be calculated to quantify the impact of the statistical heterogeneity on the meta-analysis. The Cochrane Handbook classifies the I^2^ values into 4 categories: 0% to 40%, might not be important; 30% to 60%, indicates moderate heterogeneity; 50% to 90%, represents substantial heterogeneity; 75% to 100%, suggests considerable heterogeneity. If I^2^ is more than 50%, the cause of the heterogeneity will be analyzed by meta-regression method using R software package

#### 2.3.8 Assessment of reporting biases

When more than 10 trials are included, funnel plot will be generated to observe the reported bias. Dissymmetry funnel plot indicates high risk of reporting bias, while symmetric funnel plot indicates low risk.

#### 2.3.9 Data synthesis

We will use RevMan software (V.5.3) for data synthesis, if there is no statistical heterogeneity among the results, a fixed-effects model will be used for meta-analysis. Otherwise, the heterogeneity source will be further analyzed and a random-effects model will be used for meta-analysis after excluding the effects of significant clinical heterogeneity. But when there is significant clinical heterogeneity, we will use subgroup analysis or sensitivity analysis, or only descriptive analysis.

#### 2.3.10 Subgroup analysis and investigation of heterogeneity

If data are available, a subgroup analysis will be conducted according to the type of acupressure (wristband or finger pressure), type of drugs (metoclopramide or ondansetron), time point measurement (during 0–6 hours or more than 6 hours after acupressure) and patients with different degree of risk factors (low, moderate and high). When considerable heterogeneity is detected in a previous analysis, a further subgroup analysis will be performed if necessary.

#### 2.3.11 Sensitivity analysis

Sensitivity analysis is a main way to assess the robustness and reliability of the results. If the sensitivity analysis did not substantially change the results, the reliability of the results was greatly increased. Otherwise, the results should be explained prudently. We will consider several decision nodes in the system review process to implement a sensitivity review, such as small studies, methodological weaknesses and missing data.

#### 2.3.12 Grading the quality of evidence

The quality of evidence will be assessed by Grading of Recommendations Assessment, Development and Evaluation (GRADE).^[21]^ The evidence quality will be rated as ‘high’, ‘moderate’, ‘low’, or ‘very low’ according to the GRADE rating criteria.

## 3 Discussion

Complications of PONV have significantly negative impact on the rehabilitation and life quality of patients who are undergoing surgery. Because of the side effects of antiemetic drugs, acupressure PC6 point, as one of the complementary and alternative medicine, has been widely used in clinical practice. However, the effectiveness and safety of acupressure PC6 point is still controversial. Thus, we will perform a systematic review to assess the effectiveness and safety of PC6 point acupressure in patients undergoing surgery.

The PC6 acupoint lies between the tendons of the palmaris longus and flexor carpi radialis muscles, 4 cm proximal to the wrist crease.^[22]^ Nowadays, the mechanism of acupressure PC6 point for the treatment of PONV has not been established. Different studies ^[23,24,25]^ have shown that stimulating PC6 point may affect the endocrine system of the body, regulate the level of beta-endorphins in cerebrospinal fluid and the transmission of endogenous opioids and 5-hydroxytryptamine in serum, inhibit the secretion of gastric acid, regulate gastrointestinal function, and thus stop nausea and vomit.

Previous systematic reviews have been conducted to compare the effectiveness and safety between acupressure PC6 point and sham control. But if it is evaluated by Grading of Recommendations Assessment, Development and Evaluation (GRADE),^[21]^ it exists some defects. It did not include completed trials with unpublished data.^[12,13]^ Furthermore, it did not report the funnel plot and risk of bias.^[13]^ Those defects can significantly influence the quality of evidence. Therefore, we will update this review and include unpublished trials, hoping to provide better evidence for the treatment of PONV.

There are some limitations in this review. We will not distinguish patients’ age, gender, severity of disease and anesthesia method, which may take some heterogeneity in this review. Because of the barrier of language, we will only include trials published in English or Chinese. In addition, there exists controversy to evaluate nausea, so we will not distinguish the assessment criteria of nausea, this may also carry heterogeneity.

In conclusion, to our knowledge, this study will be the first systematic review to evaluate the effectiveness and safety of acupressure on PC6 point compared with different antiemetic drugs. And we will assess the effectiveness at different measurement time points after applying acupressure. We believe that the findings of this systematic review will inform our understanding of the value of PC6 point acupressure in treating PONV. This evidence may also provide helpful evidence for treating PONV by applying acupressure in clinical practice.

## Author contributions

**Conceptualization:** Jiao Yang, Fan-rong Liang.

**Data curation:** Jiao Yang, Yilu Jiang.

**Formal analysis:** Jiao Yang, Yilu Jiang, QianHua Zheng.

**Funding acquisition:** Fan-rong Liang.

**Investigation:** Jiao Yang, Jiao Chen.

**Methodology:** Jiao Yang, Yilu Jiang, Ying Chen, Jiao Chen.

**Project administration:** Jiao Yang, Ying Chen, Mingsheng Sun.

**Resources:** Jiao Yang, Yilu Jiang, QianHua Zheng.

**Software:** Jiao Yang, Yilu Jiang, QianHua Zheng.

**Supervision:** Mingsheng Sun, Jiao Chen, QianHua Zheng, Fan-rong Liang.

**Validation:** Jiao Yang, Ying Chen, Mingsheng Sun.

**Visualization:** Jiao Yang, Yilu Jiang, Ying Chen.

**Writing – original draft:** Jiao Yang, Yilu Jiang.

**Writing – review & editing:** QianHua Zheng, Fan-rong Liang.
